# Are we correctly diagnosing and screening for bipolar depression?

**DOI:** 10.47626/1516-4446-2024-3808

**Published:** 2024-11-25

**Authors:** Adriana Munhoz Carneiro, Fernando dos Santos Fernandes, Ricardo Alberto Moreno, Doris Hupfeld Moreno

**Affiliations:** Programa de Transtornos Afetivos, Instituto de Psiquiatria, Faculdade de Medicina, Universidade de São Paulo, São Paulo, SP, Brazil

During our years treating patients in a mood disorder department, one of the most challenging problems has been the misdiagnosis of bipolar disorder (BD). Most of our patients have been referred due to difficult-to-treat depression. We have faced two important issues among these patients: 1) the misdiagnosis of BD as major depressive disorder; and 2) the lack of appropriate treatment for BD patients who do not fit within the current DSM criteria for BD or severity scales.

To illustrate these challenges, we analyzed responses from 12 outpatients from the AIUNI clinical trial who discontinued 12-week antidepressant treatment due to a re-diagnosis of BD type II.^1^ Patients were assessed using the Structured Clinical Interview for DSM-IV, which classified five as having anxious symptoms and the remaining seven as having melancholic features. The average patient age was 38.25 (SD, 10.56) years, with 50% being female (n=6).

Baseline depressive scores were moderate (HAMD = 20.91; SD = 5.45), and manic scores were very low (YMRS = 2.08; SD = 2.50). The Young Mania Rating Scale items irritability, speech, and language-thought had the highest scores (2 to 6). Only one patient reported a reduced need for sleep. Depressed mood and decreased sexual interest were frequent. However, anxiety (psychic and somatic), somatic symptoms, and reduced appetite were the most commonly scored items; half of the patients presented severe early insomnia (n=6).

Anxiety is associated with mixed symptoms, correlating with the severity of depressive symptoms during manic episodes, and vice-versa.^2^ In these terms, we emphasize the need for a deeper comprehension of patient characteristics and increased early detection of BD. Mixed symptoms, which are a crucial aspect in BD diagnosis, are rarely measured or are superficially addressed, as if they were merely a juxtaposition of two syndromes rather than a multidimensional concept.^3^

This gap in assessment could lead to a substantial number of patients remaining undetected, mirroring our Mood Disorder Department experience. As we consider the coexistence of various symptoms, we must find a way to effectively assess this complex interplay. Finally, we draw attention to the need for assessment methods to capture the full and multidimensional- spectrum of BD. Such an evolution in diagnostic strategy is vital for deeper understanding and more accurate assessment.

## Disclosure

The authors report no conflicts of interest.
